# A novel method for rapid and reliable detection of complex vertebral malformation and bovine leukocyte adhesion deficiency in Holstein cattle

**DOI:** 10.1186/2049-1891-3-24

**Published:** 2012-07-23

**Authors:** Yi Zhang, Xuehua Fan, Dongxiao Sun, Yachun Wang, Ying Yu, Yan Xie, Shengli Zhang, Yuan Zhang

**Affiliations:** 1 National Engineering Laboratory for Animal Breeding, Key Laboratory of Animal Genetics and Breeding and Reproduction of Ministry of Agriculture, College of Animal Science and Technology, China Agricultural University, Beijing, 100193, China

## Abstract

**Background:**

Complex vertebral malformation (CVM) and bovine leukocyte adhesion deficiency (BLAD) are two autosomal recessive lethal genetic defects frequently occurring in Holstein cattle, identifiable by single nucleotide polymorphisms. The objective of this study is to develop a rapid and reliable genotyping assay to screen the active Holstein sires and determine the carrier frequency of CVM and BLAD in Chinese dairy cattle population.

**Results:**

We developed real-time PCR-based assays for discrimination of wild-type and defective alleles, so that carriers can be detected. Only one step was required after the DNA extraction from the sample and time consumption was about 2 hours. A total of 587 Chinese Holstein bulls were assayed, and fifty-six CVM-carriers and eight BLAD-carriers were identified, corresponding to heterozygote carrier frequencies of 9.54% and 1.36%, respectively. The pedigree analysis showed that most of the carriers could be traced back to the common ancestry, Osborndale Ivanhoe for BLAD and Pennstate Ivanhoe Star for CVM.

**Conclusions:**

These results demonstrate that real-time PCR is a simple, rapid and reliable assay for BLAD and CVM defective allele detection. The high frequency of the CVM allele suggests that implementing a routine testing system is necessary to gradually eradicate the deleterious gene from the Chinese Holstein population.

## Background

Complex vertebral malformation (CVM) and bovine leukocyte adhesion deficiency (BLAD) are two important inherited lethal defects in dairy cattle. Both are autosomal recessive and the mutant allele can be identified by single nucleotide polymorphism. The CVM allele results from a mutation at amino acid 180 in the gene SLC35A3 on BTA3 [1] and the BLAD mutant from a missense mutation at amino acid 128 in the gene CD18 on BTA1 [2] . It was known that the intensive use of a few elite Holstein sires, e.g. Carlin-M Ivanhoe Bell (registration number US1667366, born in 1974) and his father, Pennstate Ivanhoe Star (registration number US1441440, born in 1963), who carried lethal recessive alleles of both CVM and BLAD, widely disseminated the defective alleles to the world [1,2]. CVM and BLAD are probably the two most frequent inherited defects occurring in Holstein cattle during the past decades. For instance, it was reported that the frequency of the BLAD allele reached as high as 24% in 2000, and the frequency of the CVM allele ranged from 9% to 16% during 2001 to 2007 in the German Holstein population [3]. CVM and BLAD defective alleles have also been detected in Chinese dairy populations [4-6].

Genetic defects, especially those lethal disorders like CVM and BLAD, have been one important issue in dairy cattle breeding. These defects typically cause embryonic deaths, abortions and stillborn calves, leading to negative influence on reproduction efficiency or reduced production. The development of easy and quick methods for the accurate diagnosis of mutations responsible for genetic defects would assist breeders to identify carriers and carry out a breeding program to eradicate them from the dairy population. To date, several genotyping methods [2,6-11] have been developed, but they all require several technical steps and are not easily amenable to automation or high-throughput genotyping. The real-time PCR involves a probe labeled with a reporter dye and quencher, which are cleaved during DNA amplification by Taq DNA polymerase, enabling the reporter dye to fluoresce and accumulate [12]. It has proven to be a rapid, robust, accurate, and sufficiently high-throughput technique for SNP analysis [13]. In the current study, we have developed a real-time PCR-based genotyping assay to screen the active Holstein sires to determine the carrier frequency of CVM and BLAD in Chinese dairy cattle population.

## Methods

Semen samples were collected from 587 Chinese Holstein bulls in 14 regional bull stations in China. Genomic DNA was extracted using the high-salt method [14]. Three positive control DNA templates were prepared to represent the three different genotypes. The wild type and heterozygote templates were derived from DNA samples genotyped by the previously reported restriction enzyme-based assays [2,10] and confirmed by sequencing. The template of the recessive homozygous genotype, however, was generated by cloning PCR products of a heterozygote using the TA cloning kit according to the manufacturer’s instructions (Invitrogen, San Diego, CA, USA).

Amplifications were performed on a Loche LightCycler® 480 real-time PCR system (Roche Applied Science, Penzberg, Germany) using TaqMan probes and specific primer pairs (Table 1) which were synthesized by Applied Biosystems, Foster City, USA. Two sets of primer pairs and probes were designed based on the published sequences of the CD18 (GenBank Accession Number Y12672) and SLC35A3 gene (GenBank Accession Number AY160683), respectively. In each set of probes, one probe which perfectly matched the mutant sequence variant was 5’-labeled with 6-carboxyfluorescein (FAM); another probe which matched the wild type sequence variant was 5’-VIC labeled, and both probes had a non-fluorescent quencher and a minor groove binding moiety (MGB).

**Table 1:** Primer and probe sequences, dual labeling (only for probes), and position in the reference sequences in real-time PCR-based assays for CVM and BLAD

**Genetic defect**	**Molecule**	**Oligo sequence, 5′ to 3′**	**Reporter, 5′**	**Quencher, 3′**	**Position**^**1**^
CVM					
	Primers				
	SLC35A3-FWD	AGCTGGCTCACAATTTGTAGGT			9840-9861
	SLC35A3-REV	CTCAAAGTAAACCCCAGCAAAGC			9896-9918
	Probes				
	SLC35A3-WD	TCATGGCAGTTCTCA	VIC	NFQ	9863-9877
	SLC35A3-MUT	TCATGGCATTTCTCA	FAM	NFQ	9863-9877
BLAD					
	Primers				
	CD18-FWD	GTTGCGTTCAACGTGACCTT			1154-1173
	CD18-REV	GAGTAGGAGAGGTCCATCAGGTA			1208-1230
	Probes				
	CD18-WD	CCCCATCGACCTGTAC	VIC	NFQ	1192-1207
	CD18-MUT	CCCATCGGCCTGTAC	FAM	NFQ	1193-1207

^1^Positions are based on the published sequences of the CD18 (GenBank Accession Number Y12672) and SLC35A3 gene (GenBank Accession Number AY160683) for BLAD and CVM, respectively.

Two independent real-time PCR reactions were carried out for each sample to determine the genotype of the CD18 and SLC35A3 loci, respectively. A 10 μl reaction consisted of 0.25 μl 40 × SNP genotyping assay mix (including primers and probes) and 5 μl 2 × TaqMan universal PCR master mix, 1 μl of genome DNA (5-20 ng), and 3.75 μl double-distilled H_2_O. PCR conditions were 95 °C for 10 minutes followed by 50 cycles of 95 °C for 15 seconds and 60 °C for 1 minute.

## Results and discussion

**Figure 1:**
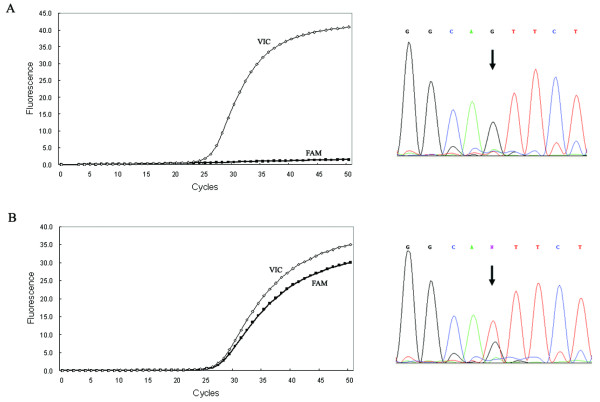
**Real-time polymerase chain reaction (PCR) amplification plot of (A) the wild type homozygote and (B) the carrier of CVM gene, and confirmed by direct sequencing**. FAM-labeled probe is complementary to mutant allele and VIC-labeled probe is complementary to wild type allele. Heterozygous position is indicated by arrow.

**Figure 2:**
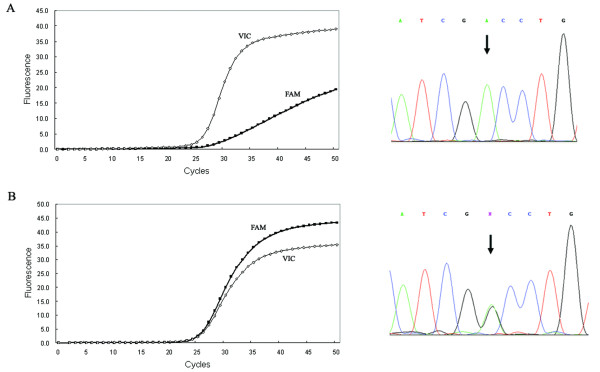
**Real-time polymerase chain reaction (PCR) amplification plot of (A) the wild type homozygote and (B) the carrier of BLAD gene, and confirmed by direct sequencing**. FAM-labeled probe is complementary to mutant allele and VIC-labeled probe is complementary to wild type allele. Heterozygous position is indicated by arrow.

Allelic discrimination can be performed by analyzing the real-time amplification plots. In theory, the wild-type probes will only hybridize with the wild type and produce a typical amplification curve from the VIC signal channel, while the mutant probes hybridize only with mutant target and generate an amplification curve from the FAM signal channel. Thus, genotype can be accurately determined by comparing the amplification curves, as shown in Figure 1. When real-time amplification plots were examined for BLAD, a weak non-specific signal was observed in the wild type allele (Figure 2). This phenomenon was also reported by a previous study [15]. It happens probably because the allele-specific probe has only single base mismatch with the other allele; and when the nucleotide sequence near the SNP site is highly rich in G/C or it contains certain sequence combinations, the probe tend to be less discriminative to the mismatched allele. However, the pattern of the real-time amplification plots can still be easily differentiated between the wild type and mutant, because intensity of the non-specific signal is much lower than target signal.

In all, 587 Chinese Holstein bulls were assayed using the newly developed method. Fifty-six CVM-carriers and eight BLAD-carriers were identified, corresponding to the heterozygote carrier frequency 9.54% and 1.36%, respectively; and no mutant homozygote was found. One bull was detected as a carrier of both BLAD and CVM.

**Figure 3:**
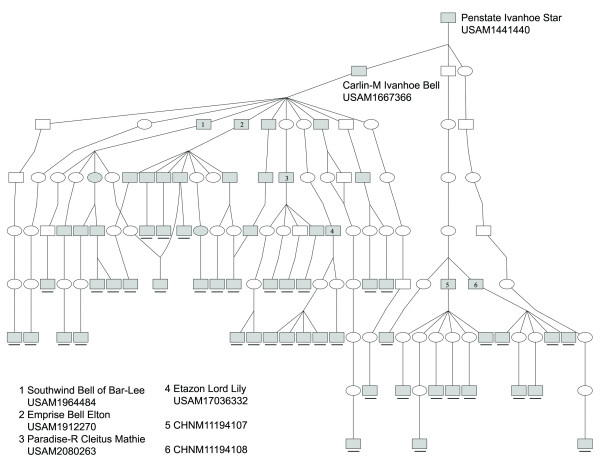
**Pedigree network of CVM carrier sires**. It was constructed using Pedigraph software [16]. (□ male without genotype, ○female without genotype, ■male carrier, ● female carrier. The carriers which were identified in current study are underlined).

**Figure 4:**
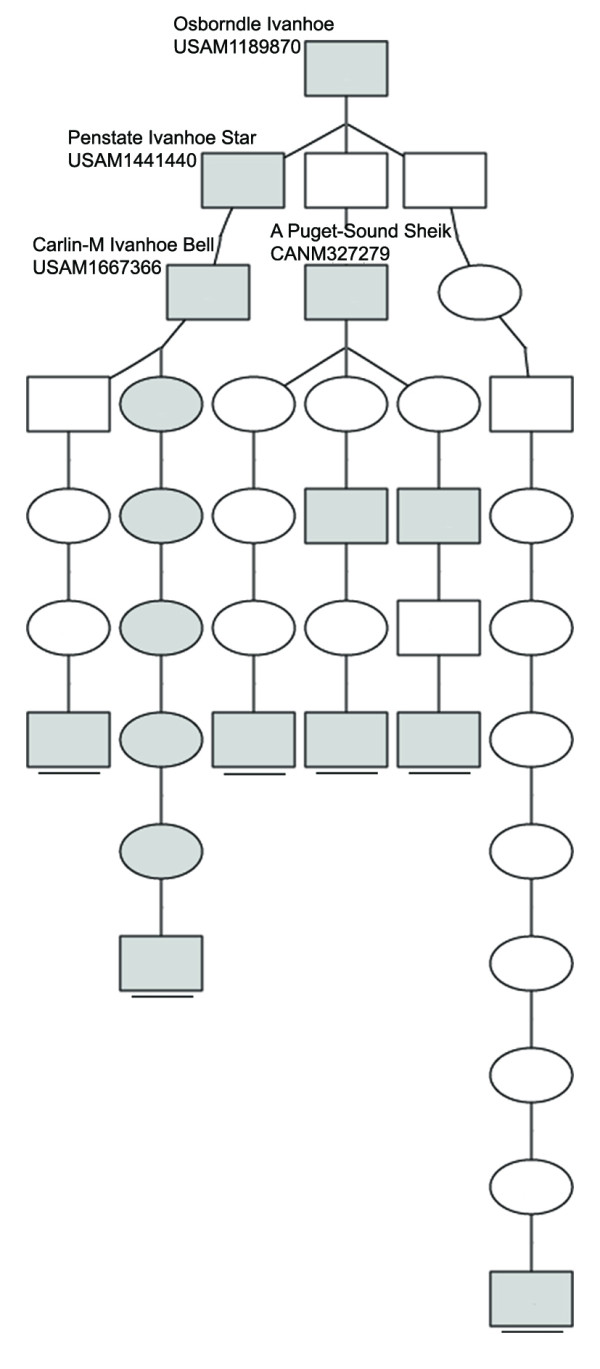
**Pedigree network of BLAD carrier sires**. It was constructed using Pedigraph software [16]. (□ male without genotype, ○female without genotype, ■male carrier, ● female carrier. The carriers which were identified in current study are underlined).

Pedigree analysis were carried out to trace the ancestries of the carriers using the pedigree databases of Holstein cattle of China (http://www.holstein.org.cn/), USA (http://www.holsteinusa.com/), Canada (https://www.holstein.ca/), and Australia (http://www.holstein.com.au/). Results showed that 42 of 56 CVM-carriers were traced back to the common ancestor, the elite US sire Pennstate Ivanhoe Star. His son, Carlin-M Ivanhoe Bell, however, is the most responsible bull for the spread of CVM lethal allele (29/42) (Figure 3). His prominent offsprings, including Elton, Southwind and Mathie, and Lord Lily, were also heterozygous for the gene. In Chinese domestic bulls, two elite sires (CHN11194107 and CHN11194108) play important roles in the spread of CVM allele in China. In the case of BLAD, six of eight carriers were traced back to Osborndale Ivanhoe (Figure 4). In addition to the two elite US sires, Penstate Ivanhoe Star and Carlin-M Ivanhoe Bell, the Canada sire, A Puget-Sound Sheik, was the critical sire responsible for BLAD prevalence.

The correctness of real-time genotyping was evaluated by direct sequencing (Figures 1 and 2). All identified carriers and thirty randomly selected non-carriers were sequenced. Results showed no discrepancies between the two assay strategies. These results demonstrated that real-time PCR is a reliable assay for genotyping the BLAD and CVM loci.

Several molecular methods, including PCR-RFLP [2,7] PCR-PIRA [10], AS-PCR [8,9] and SSCP [6,11] have been developed and proved useful for screening of BLAD or CVM in practice. Generally, these approaches are relatively cost-effective and easy to use because only basic equipment and reagent are involved. However, they all involve several technical steps and are time-consuming (e.g. using SSCP [9] to screen CVM carrier in our lab required minimum 7-8 hours after DNA extraction). The real-time PCR-based assay developed here, however, required only one amplification step to obtain results and the time involved was about 2 hours after DNA extraction from the sample. There was no post-PCR handling required, which reduced the risk of carry-over contamination. These advantages allow the real-time PCR assay to be more amenable to high-throughput sample processing.

## Conclusions

The real-time PCR-based method for BLAD and CVM carrier detection is simple, rapid, reliable, and ready for high-throughput genotyping. The high frequency of the CVM allele found here suggests that implementing a routine testing system using our novel method is more than necessary. All AI sires could be screened, efficiently allowing a breeding program to gradually eradicate these deleterious genes from the Chinese Holstein population.

## Abbreviations

CVM, Complex vertebral malformation; BLAD, Bovine leukocyte adhesion deficiency; BTA, Bos taurus chromosome; CD18, Integrin beta-2; SLC35A3, Solute carrier family 35 (UDP-N-acetylglucosamine (UDP-GlcNAc) transporter), member A3.

## Competing interests

The authors declare that they have no competing interests.

## Authors’ contributions

YZ designed the study, analyzed the data and prepared the manuscript. XF developed the new genotyping method and prepared the manuscript. DS participated in the design of the study. YW participated in the preparation and the revision of the manuscript. YY and YX contributed to the sample collection and genotyping. YZ and SZ were the project leaders and contributed to project design, sample collection, result interpretation and manuscript preparation. All authors read and approved the manuscript.
